# Cascading effects of hypobaric hypoxia on the testis: insights from a single-cell RNA sequencing analysis

**DOI:** 10.3389/fcell.2023.1282119

**Published:** 2023-11-15

**Authors:** Yun-Hua Ji, Lin-Meng Wang, Fu-Xun Zhang, Hao-Zhong Hou, Zhi-Rong Luo, Qi Xue, Man-Man Shi, Yong Jiao, Dong Cui, Da-Li He, Wei Xue, Yu-qi Wen, Qi-Sheng Tang, Bo Zhang

**Affiliations:** ^1^ Department of Urology, Tangdu Hospital, Air Force Military Medical University, Xi’an, Shanxi, China; ^2^ Department of Urology, Xijing Hospital, Air Force Military Medical University, Xi’an, Shanxi, China; ^3^ Department of Bioinformatics, Institute of Health Service and Transfusion Medicine, Beijing, China

**Keywords:** cell atlas, cell type, hypobaric, hypoxia, single-cell RNA sequencing, testis

## Abstract

Most mammals tolerate exposure to hypobaric hypoxia poorly as it may affect multiple regulatory mechanisms and inhibit cell proliferation, promote apoptosis, limit tissue vascularization, and disrupt the acid–base equilibrium. Here, we quantified the functional state of germ cell development and demonstrated the interaction between the germ and somatic cells via single-cell RNA sequencing (scRNA-seq). The present study elucidated the regulatory effects of hypobaric hypoxia exposure on germ cell formation and sperm differentiation by applying enrichment analysis to genomic regions. Hypobaric hypoxia downregulates the genes controlling granule secretion and organic matter biosynthesis, upregulates tektin 1 (TEKT1) and kinesin family member 2C (KIF2C), and downregulates 60S ribosomal protein 11 (RPL11) and cilia- and flagella-associated protein 206 (CFAP206). Our research indicated that prosaposin-G protein-coupled receptor 37 (PSAP-GPR37) ligands mediate the damage to supporting cells caused by hypobaric hypoxic exposure. The present work revealed that hypoxia injures peritubular myoid (PTM) cells and spermatocytes in the S phase. It also showed that elongating spermatids promote maturation toward the G2 phase and increase their functional reserve for sperm–egg binding. The results of this study provide a theoretical basis for future investigations on prophylactic and therapeutic approaches toward protecting the reproductive system against the harmful effects of hypobaric hypoxic exposure.

## 1 Introduction

The plateau areas at altitudes >3,000 m have distinctive physical and climatic attributes, including wide diurnal temperature variation, low atmospheric pressure (hypobaria), low oxygen concentration (hypoxia), and intense solar radiation. These factors may exert various adverse effects on humans (Alsup et al., 2019). The inhabitants of these regions undergo certain negative physiological and structural transformations, including decreased maximal oxygen consumption, compromised cognitive function, and disruptions in sleep patterns (Beretta et al., 2017). The low atmospheric pressure and oxygen levels characteristic of these regions may be responsible for these undesirable changes (Viscor et al., 2018). The effects of this environment on the respiratory and circulatory systems have been the subjects of numerous prior investigations. Nevertheless, its impact on the human reproductive system is poorly understood. In mammals, hypoxia may damage cellular-level regulatory mechanisms (Oyedokun et al., 2023). The low atmospheric oxygen levels characteristic of high altitudes are detrimental to male fertility (He T. et al., 2021). Each type of hypoxia has a distinct impact on spermatogenesis (He H. et al., 2022). Although acute hypoxia adversely affects male fertility within minutes, the negative impact of chronic hypoxia on sperm production may not be apparent until several days after the onset of exposure. However, both acute and chronic hypoxia that may occur at different altitudes may cause abnormal spermatogenesis (Reyes et al., 2012; He et al., 2015). In male germ cell development, post-meiotic cells are particularly vulnerable to hypoxia-induced damage. Prior animal and experimental studies have empirically demonstrated the association between hypoxia and impaired fertility (Zepeda et al., 2014; Moore, 2021).

Spermatogenesis is the intricate process of sperm cell formation that occurs within the seminiferous epithelium of the testis. A male germline stem cell differentiates into a spermatogonial cell, which, in turn, multiplies and undergoes mitotic, meiotic, and morphological transformations and ultimately matures into a spermatocyte (Bose et al., 2015). The seminiferous tubules comprise 60%–80% of the total volume of the testis and contain germ, peritubular, and Sertoli cells (Kumar and Singh, 2021). The distinct spermatogonial stem cells within the specialized cellular framework of the varicocele drive spermatogenesis (Chen et al., 2021). Traditional bulk sequencing methods have been ineffective at clarifying the response patterns of the different cell types implicated in spermatogenesis. For this reason, the molecular and regulatory mechanisms of sperm differentiation and abnormal sperm production were poorly understood. The recent advent of single-cell RNA sequencing (scRNA-seq) technologies has substantially enriched our comprehension of various cellular mechanisms (Zhao et al., 2020). These methods have facilitated the precise investigation of individual cell populations within the testis. Cap analysis of gene expression (CAGE) is a high-throughput gene expression profiling technique that exposes the heterogeneity in gene expression among the various developmental stages of germ cells. CAGE unveils the transcriptome characteristics and heterogeneity of cells with the same morphological type. Hence, it could improve the identification and classification of different cell populations (Lukassen et al., 2018). Previous studies analyzed and compared the transcriptomes of various testicular cell types. However, they did not comprehensively explore rare cell subpopulations or genes that are differentially expressed across cell types. Here, we used single-cell RNA-seq to quantify the functional state of germ cell development and demonstrate the interactions among germ and somatic cells. The results of this work may help explain the relationship between hypoxia and testicular injury.

## 2 Materials and methods

### 2.1 Construction of the testis injury model

The experimental procedure and protocol used in the present study were approved (No. 2913) by the Animal Center of Air Force Medical University, Shaanxi, China, and performed in accordance with the National Guidelines for the Care and Use of Experimental Animals. Six-week-old male C57BL/6 mice (Experimental Animal Center of Air Force Military Medical University, Shaanxi, Xi’an, China) were randomly allocated either to the control group (n = 10) exposed to a normal environment for 4 weeks or the hypoxia exposure group (n = 10) exposed to hypobaric hypoxia for 4 weeks. All mice were fed a standard diet, had *ad libitum* food and water access, and were subjected to a 12 h day/12 h night photoperiod. The mice in the hypoxia exposure group were placed and maintained in a hypobaric (low-pressure) hypoxia chamber (PaO_2_ = 14 kPa; 14.5%) for 4 weeks. These conditions simulated 5,000 m altitude.

### 2.2 Sample collection and processing

**FIGURE 1:**
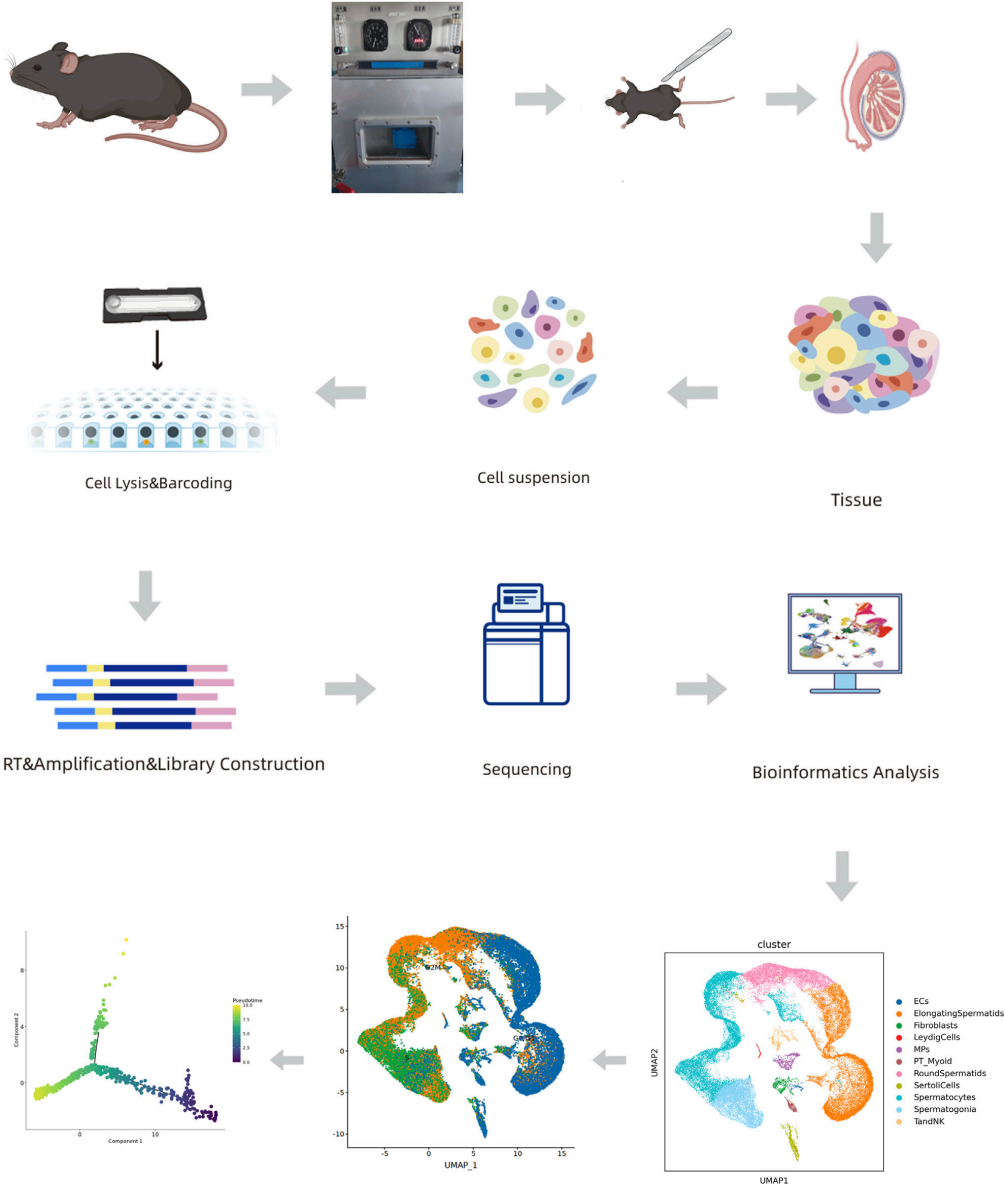
Process flow diagram.

The surviving mice were randomly selected from each group (n = 3) and subjected to single-cell RNA-seq. The mice were euthanized, and their testes were excised. The testes were washed thrice with phosphate-buffered saline (PBS; pH 7.4). Fresh testicular tissues were stored in the GEXSCOPE™ tissue preservation solution (Singleron Biotechnologies, Cheshire, CT, United States), placed on ice within 30 min after surgery, and processed into single-cell suspensions (Figure 1), according to a previously reported procedure (Rong et al., 2022).

### 2.3 Singleron Matrix™ single-cell RNA sequencing

Single-cell suspensions were prepared at a density of 1 × 10^5^/mL in PBS (Thermo Fisher Scientific, Waltham, MA, United States). Single-cell suspensions were then loaded onto microfluidic devices. The scRNA-seq libraries were constructed with a GEXSCOPE^®^ Single-Cell RNA Library Kit, according to the Singleron GEXSCOPE^®^ protocol (Singleron Biotechnologies). The individual libraries were diluted to 4 nM, pooled, and sequenced as 150-bp paired-end reads on the Illumina NovaSeq platform (Illumina, San Diego, CA, United States).

### 2.4 Primary raw read data analysis

Raw scRNA-seq reads were processed on an internal pipeline with fastQC v. 0.11.4 (https://www.bioinformatics.babraham.ac.uk/projects/fastqc/) (de Sena Brandine and Smith, 2019) and fastp (https://github.com/OpenGene/fastp) (Chen et al., 2018) to remove low-quality reads and with Cutadapt (https://cutadapt.readthedocs.io/en/stable/) to trim poly-A tail and adapter sequences. In this manner, gene expression matrices were generated. Cell barcodes and unique molecular identifiers (UMIs) were then extracted. STAR v. 2.5.3a (https://github.com/alexdobin/STAR) (Dobin et al., 2013) was then used to map the reads to the reference genome GRCm38 (Ensembl v. 92 annotation; https://www.ncbi.nlm.nih.gov/datasets/genome/GCF_000001635.20/). The UMI and gene counts were acquired for each cell via featureCounts v. 1.6.2 (https://www.rdocumentation.org/packages/Rsubread/versions/1.22.2/topics/featureCounts) (Liao et al., 2014) and used to generate expression matrix files for the subsequent analysis.

### 2.5 Quality control, dimension reduction, and clustering

Scanpy v. 1.8.1 (https://pypi.org/project/scanpy/) under Python v. 3.7 (https://www.python.org/downloads/) was used for quality control, dimensionality reduction, and clustering. The expression matrix was filtered for each sample dataset, according to the following exclusion criteria: 1) cells with gene count <200 and the top 2% gene counts; 2) cells with the top 2% UMI counts; 3) cells with mitochondrial content >10%; and 4) genes expressed in fewer than 5 cells (Satija et al., 2015; Baladehi et al., 2022). After filtering, 49,027 cells were retained for the downstream analyses, and there were averages of 2,146 genes and 8,911 UMIs/cell. The raw count matrix was normalized by the total counts/cell and logarithmically transformed into a normalized data matrix. The top 2,000 variable genes were selected by setting flavor = ‘seurat’ using Seurat v 4.0.0 (https://satijalab.org/seurat) (Korsunsky et al., 2019). A principal component analysis (PCA) was performed on the scaled variable gene matrix, and the top 20 principal components were used for clustering and dimensional reduction. The Louvain algorithm (https://neo4j.com/docs/graph-data-science/current/algorithms/louvain/) was used at 1.2 resolution to separate the cells into 26 clusters, which were then visualized by Uniform Manifold Approximation and Projection (UMAP).

### 2.6 Differentially expressed gene (DEG) analysis

The FindMarkers function in Seurat (https://satijalab.org/seurat/reference/findmarkers), based on the Wilcox likelihood-ratio test with its default parameters, was applied to identify differentially expressed genes (DEGs). Those that were expressed in >10% of all cells in a cluster and had an average log fold change (FC) > 0.25 were designated DEGs. The expression of the canonical markers in the DEGs was combined with literature data to annotate the cell types in each cluster. Marker expression in each cell type was displayed using heatmaps, dot plots, and violin plots generated by the DoHeatmap/DotPlot/Vlnplot function in Seurat (https://satijalab.org/seurat).

### 2.7 Pathway enrichment analysis

Gene Ontology (GO) and Kyoto Encyclopedia of Genes and Genomes (KEGG) analyses were performed via the “clusterProfiler” package in R (https://bioconductor.org/packages/release/bioc/html/clusterProfiler.html) (Yu et al., 2012) to investigate the potential DEG functions. Pathways with adjected *p* < 0.05 were deemed significantly enriched. The molecular function (MF), biological process (BP), and cellular component (CC) categories in GO served as references.

### 2.8 Cell-type annotation

#### 2.8.1 Cell-type recognition with Cell-ID

Cell-ID is a multivariate approach that extracts gene signatures for each cell and recognizes cell identities via hypergeometric tests (HGTs). Multiple correspondence analysis was used to reduce the dimensionality of the normalized gene expression matrix. Cells and genes were projected onto the same low-dimensional space. Gene rankings were calculated, and the most abundantly featured gene sets were identified for all cells. HGT was performed on these gene sets and compared against the brain reference from the SynEcoSys database (https://www.synecosys.com/#/), which contains the featured genes of all cell types. Cell types were distinguished based on their minimum HGT *p*-values. The frequency of each cell type in each cluster was calculated, and each cluster was identified according to its most abundant cell type.

The cell types in each cluster were identified based on the expression of their canonical markers in the SynEcoSys™ reference database (Singleron Biotechnology). SynEcoSys™ has collections of canonical cell-type markers for single-cell sequencing data derived from the CellMarker 2.0 database (http://bio-bigdata.hrbmu.edu.cn/CellMarker), PanglaoDB (https://panglaodb.se).

#### 2.8.2 Subtyping of major cell types

High-resolution mapping of elongating spermatids, round spermatids, spermatocytes (Qiu et al., 2017), spermatogonia, and Sertoli cells was performed by extracting and reclustering according to the aforementioned procedures and setting the clustering resolution to 0.6 (Bergen et al., 2020).

### 2.9 Batch effect removal

The batch effect between the samples was removed using Harmony v. 1.0 (https://github.com/immunogenomics/harmony) and the top 20 principal components obtained from the PCA.

### 2.10 Cell doublet filtering

Cell doublets were estimated based on the canonical cell marker expression patterns. Clusters enriched with multiple cell type-specific markers were excluded from the downstream transcription factor (TF) regulatory network analysis (Aibar et al., 2017).

### 2.11 Cell cycle analysis

The cell cycle score was calculated for each cell via the CellCycleScoring function in Seurat v. 3.1.2 (https://satijalab.org/seurat/articles/cell_cycle_vignette) (Shannon et al., 2003).

### 2.12 Cell–cell interaction analysis: CellPhoneDB

Cell–cell interactions (CCIs) among elongating spermatids, round spermatids, spermatocytes, spermatogonia, and Sertoli cells were predicted with CellPhoneDB v. 2.1.0 (https://github.com/Teichlab/cellphonedb) and based on known ligand–receptor pairs. A total of 1,000 permutations were used to calculate the null distribution of the average ligand–receptor pair expression in randomized cell identities. The thresholds for individual ligand and receptor expression were set using a cutoff based on the distribution of the average log expression of all genes across each cell type. The predicted interaction pairs with *p* < 0.05 and average log expression >0.1 were considered significant and were visualized with the heatmap_plot and dot_plot functions in CellPhoneDB.

### 2.13 Pseudotime trajectory analysis: Monocle 2

The differentiation trajectories of the monocyte subtypes were reconstructed in Monocle 2 v. 2.10.0 (http://cole-trapnell-lab.github.io/monocle-release/docs/#installing-monocle). The top 2,000 highly variable genes were selected with the FindVariableFeatures in Seurat v. 3.1.2. Dimension reduction was performed in DDRTree (https://cran.r-project.org/web/packages/DDRTree/index.html) to construct the trajectory. The latter was then visualized with the plot_cell_trajectory function in Monocle 2.

### 2.14 Transcription factor regulatory network analysis (pySCENIC)

A transcription factor network was constructed with pySCENIC v. 0.11.0 (https://pyscenic.readthedocs.io/en/latest/), the scRNA expression matrix, and TFs in AnimalTFDB (https://ngdc.cncb.ac.cn/databasecommons/database/id/8). GRNBoost2 (https://github.com/aertslab/GRNBoost) was used to predict a regulatory network based on regulator and target co-expression. Then, cisTarget (https://resources.aertslab.org/cistarget/databases/) was applied to exclude indirect targets and search TF-binding motifs. AUCell (https://www.bioconductor.org/packages/release/bioc/html/AUCell.html) was then used to quantify the regulon activity in each cell. Cluster-specific TF regulons were identified according to their regulon specificity scores, and their activity was visualized using heatmaps.

### 2.15 Statistics and repeatability

The cell-type distributions between the groups were determined via unpaired two-tailed Wilcoxon rank-sum tests. The gene expression levels and gene signatures in the cells were compared between the groups via unpaired two-tailed Student’s *t*-tests. The cell distributions in paired group1 and paired group2 were performed via paired two-tailed Wilcoxon rank-sum tests. All statistical analyses and presentations were performed in R (R Core Team, Vienna, Austria). The statistical tests illustrated in the figures are shown in the legends, and *p* < 0.05 indicated statistical significance. The exact values of *n* are indicated in the figures, and *n* is defined in the figure legends.

### 2.16 Immunofluorescence and hematoxylin–eosin staining

Fresh testicular tissue was fixed in 4% (v/v) paraformaldehyde (PFA; Wuhan Servicebio Technology Co. Ltd., Wuhan, China) for 30 h, dehydrated, permeabilized, embedded in paraffin, cut into 4-μm sections, and stained with hematoxylin and eosin (HE). Frozen tissue sections that were 10-μm-thick were fixed in 4% (v/v) PFA at 20°C–25°C for 15 min in preparation for immunofluorescence staining. The peritubular myoid (PTM) cells underwent immunofluorescence staining with anti-α-SMA (No. GB11364, 1:1,000; Wuhan Servicebio Technology Co. Ltd.), anti-MYH11 (No. GB111220, 1:1,000; Wuhan Servicebio Technology Co. Ltd.), and anti-SM22α (No. GB11366, 1:1,000; Wuhan Servicebio Technology Co. Ltd.), and nuclear staining with 4′,6-diamidino-2-phenylindole (DAPI; No. G1012, 1:1,000; Wuhan Servicebio Technology Co. Ltd.).

### 2.17 Western blot analysis

At the end of the study, mice from each treatment group were euthanized, and their testes were excised and lysed in radioimmunoprecipitation assay (RIPA) buffer (Beyotime Biotechnology, Shanghai, China) containing a protease inhibitor cocktail (Sigma-Aldrich Corp., St. Louis, MO, United States). The total protein concentration was determined with a bicinchoninic acid (BCA) protein assay kit. An amount of 60 µg of lysate were fractionated by sodium dodecyl sulfate–polyacrylamide gel electrophoresis (SDS-PAGE) and transferred to polyvinylidene fluoride (PVDF) membranes. The latter were then blocked with 5% (w/v) nonfat milk at room temperature for 1 h and incubated with anti-TNF-α (Wuhan Servicebio Technology Co. Ltd.), anti-TNFR Ⅰ (No. AF0282; Affinity Biosciences, Cincinnati, OH, United States), anti-caspase-3 (No. AF6311; Affinity Biosciences), anti-ATM (No. AF4119; Affinity Biosciences), anti-Bax (No. AF0120; Affinity Biosciences), or anti-actin (No. AF7018; Affinity Biosciences) at 4°C overnight. The membranes were washed at least thrice with Tris-buffered saline with Tween 20 (TBST), subjected to horseradish peroxidase (HRP)-conjugated secondary antibody (Wuhan Servicebio Technology Co. Ltd.), and examined with an ultrasensitive enhanced chemiluminescence (ECL) detection kit (No. ZD310A; Beijing Zoman Biotechnology Co. Ltd., Beijing, China). The strip density was determined using ImageJ (National Institutes of Health (NIH), Bethesda, MD, United States), and actin-β (ACTB) was the loading control.

### 2.18 Cell culture and treatment

Sertoli cells (Pricella, China) were cultured for 4 days, set in a 5% CO_2_ + N_2_ triple gas incubator at 34°C and 1% (v/v) O_2_, incubated for 24 h and 48 h, and their corresponding protein concentrations were measured. They were also incubated with 500 ng/mL recombinant prosaposin (rPSAP) (MedChemExpress, Monmouth Junction, NJ, United States) for 30 min and subjected to Western blot analysis.

### 2.19 Statistical analysis

DEGs between the groups and cell types were identified by a Wilcoxon rank-sum test, and differences between the groups were deemed statistically significant at *p* < 0.05.

## 3 Results

### 3.1 ScRNA-seq analysis comprehensively identified the testicular cell types affected by hypobaric hypoxic exposure

**FIGURE 2:**
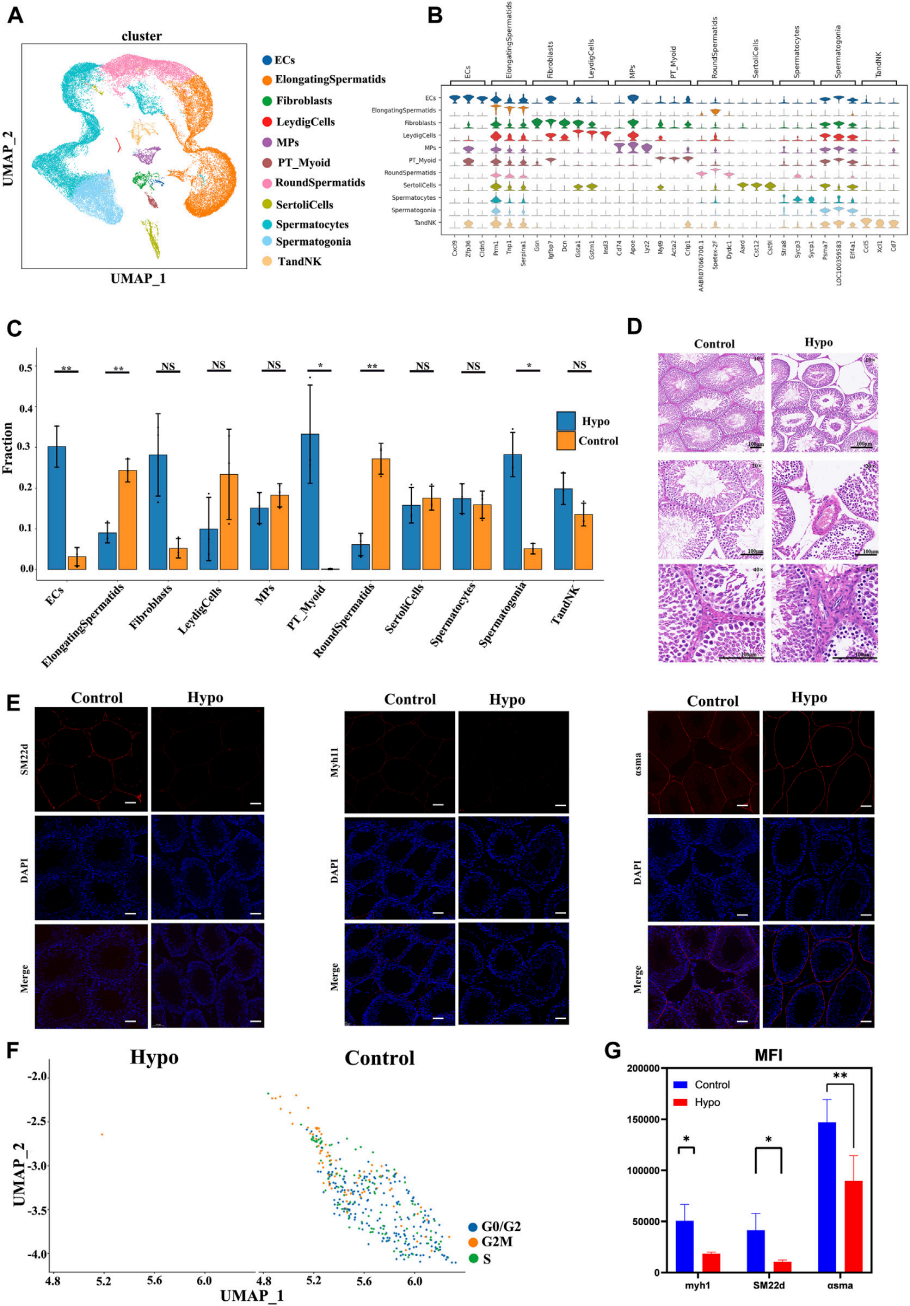
Cell lineage analyses of treated and untreated mouse testes by scRNA-seq technology. **(A)** Six distinct testicular cell populations were identified by a UMAP plot. Each color represents a unique cell subcluster. **(B)** Violin plot showing selected marker genes for each cell cluster. Different colors represent unique marker genes associated with each cell type. **(C)** Bar plot displaying the relative proportions of testicular cell clusters from treated and control mice. Blocks represent distinct subjects, and the cell quantity is proportional to block height. **(D)** Hematoxylin–eosin (H&E) staining disclosing relatively more seminiferous tubules, fewer spermatogonia, spermatogenic cell edema, and minimal inflammatory cell infiltration in the treated testes compared to the control testes. Scale bar = 100 μm. **(E)** Fluorescence microscopic images of peritubular myoid (PTM) cells. **(F)** UMAP plots of PT_Myoid. **(G)** Mean fluorescence intensity (Mean) = total fluorescence intensity (IntDen)/# tubes. Data are means ± standard deviation (S.D.) (n = 3). *p < 0.05, **p < 0.01. Scale bar = 100 μm.

We used scRNA-seq to compare the molecular events that occurred in the testes of untreated mice and those subjected to hypobaric hypoxic conditions. We obtained 49,000 cells, and the cellular viability rates for the treatment group were 93.79%, 91.42%, and 95.49%. We then compared all the samples from the treatment group against those from the control group. Figure 2A shows that the cell/gene expression matrix was visualized with UMAP for dimension reduction. The published expression patterns for marker genes helped identify certain cell types, and we annotated the clusters into 11 cell populations, accounting for nearly all known cell types in testes subjected to hypobaric hypoxic conditions (Figure 2A). These included endothelial cells (ECs), elongating spermatids (ElongatingSpermatids), fibroblasts, Leydig cells (LeydigCells), mononuclear phagocytes (MPs), peritubular myoid cells (PT_Myoid), round spermatids (RoundSpermatids), Sertoli cells (SertoliCells), spermatocytes, spermatogonia, and T and NK cells (TandNK). Numerous cell types are identified by multiple marker genes with expression patterns such as those shown in Figure 2B. Figure 2C shows that the proportions of elongating spermatids, spermatocytes, and round spermatids significantly differed from those of the other cell types. The distributions of the cell populations in the control and treatment groups were merged by UMAP to determine the impact of the hypobaria hypoxia treatment on the cell population and transcriptional changes (Supplementary Figure S1). Overall, the treatment significantly reduced the proportion and number of all cell populations, particularly those of the germ cells. Hence, hypobaric hypoxic exposure is toxic to spermatogenesis (Figure 2C). We then observed testicular sections under a light microscope (Figure 2D) and found that adjacent seminiferous tubules had large intertubular spaces. Spermatogonia were remote from the basement in most parts of the hypoxic testes. The latter also presented degraded luminal borders, no mature germ cells, and indistinct luminal borders on the germinal epithelium.

The arrest of spermatogenesis and vacuolization of the Sertoli cells was evident in the seminiferous tubules of the mice exposed to hypobaria hypoxia but not in those of the untreated mice. Immunofluorescence staining of alpha-smooth muscle actin (α-SMA), SM22d, and myosin heavy chain 11 (Myh11) was abnormal in the testes of the treated mice. We divided the average fluorescence intensity by the average background fluorescence intensity to calculate the normalized average fluorescence intensity. We then divided the total fluorescence intensity by the number of tubes to calculate the normalized average fluorescence intensity. The mean fluorescence intensity was weaker in the treatment group than that in the control. The foregoing results were consistent with those obtained by scRNA-seq (Figure 2E **–**2G). We used Western blotting to measure the protein expression levels of the cytokines implicated in the relevant signaling pathways and elucidate the apoptosis induction mechanism. Figures 2C, D show that ataxia–telangiectasia mutated (ATM), Bax, tumor necrosis factor-alpha (TNF-α), TNFR Ⅰ, and caspase-3 were all downregulated. We then identified TFs with the highest recombination signal sequence scores per cell type (Supplementary Figure S5). ETV5 in Sertoli cells maintains blood–testis barrier integrity (Yang et al., 2021). RXRG in Leydig cells is a rexinoid receptor that regulates cell differentiation (Tang et al., 2022). DMRT1 maintains consistent numbers of spermatogonia within the testis (Wei et al., 2021). We then investigated how hypobaric and hypoxic exposure affects each cell type during testicular development.

### 3.2 Post-treatment transcriptional regulation of germ cell maintenance

**FIGURE 3:**
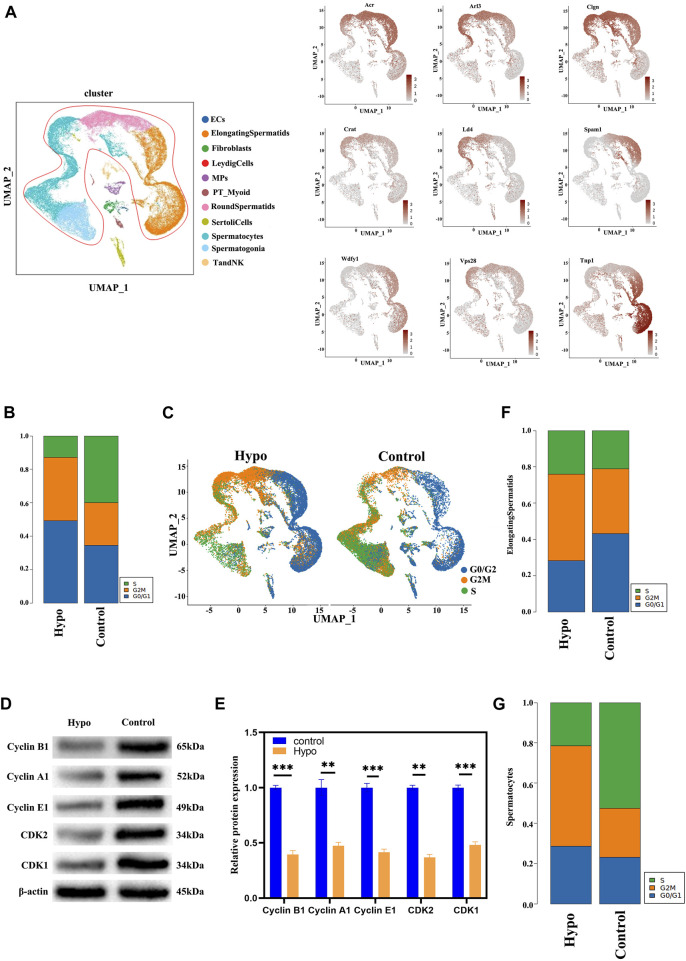
Testicular cell cluster identification by scRNA-seq. **(A)** Testicular cell cluster annotation and representative marker gene detection by UMAP visualization. **(B)** Bar graphs showing proportions of cells in various cell cycle phases. **(C)** UMAP plot of contrast pictures of the cell cycle. **(D)** Protein expression levels of cyclin A1, cyclin-dependent kinase 2 (CDK2), cyclin E1, CDK1, and cyclin B1 were significantly lower in the testes of the treated mice than in those of the control. **(E)** Relative densitometric analysis of protein bands. Data are means ± S.D. n = 3/group. ***p < 0.01, **p < 0.05 vs control. **(F)** Bar graphs showing proportions of elongating spermatids in various cell cycle phases. **(G)** Bar graphs showing proportions of spermatocytes in various cell cycle phases.

We used a single-cell dataset to validate previously reported molecules associated with testicular damage in response to hypobaric hypoxia exposure (Figure 3A). For the single-cell data, we used bar graphs to plot the DEGs between groups (Supplementary Figure S2). The results suggested that most of the testicular damage associated with hypobaria hypoxia exposure consisted of sperm cell injury. Hence, we directed our subsequent analyses toward the changes that occur in the sperm cells in response to hypobaria hypoxia exposure. The cell cycle modulates the proliferation of normal cells. Our cell cycle analysis (Figure 3B) shows that there were relatively more cells in the S and G2M phases in the treatment group than in the control. We investigated the underlying mechanisms by observing various testicular cells and mapping their subpopulations. There was wider variation in the long-type and other spermatocytes than in the other cell types (Figures 3B, C). Previous reports demonstrated that external factors may damage sperm cells at various stages of the germinal cycle. We used the data to characterize the genes implicated in the spermatogenic cycle (Supplementary Figure S3) and found that they clustered mainly in the elongated spermatocyte fraction. A subsequent cell cycle analysis corroborated these findings and revealed that most of the spermatocytes in the testes of the hypobaria/hypoxia-treated mice were in the G2 phase. Thus, hypoxia exposure may trap the spermatocytes in the S phase (Figures 3D–G). Prior studies might have underestimated the extent of the damage that hypoxia causes to elongated and other spermatocytes.

### 3.3 Effects of hypobaria and hypoxia on spermatocytes and elongating spermatids

**FIGURE 4:**
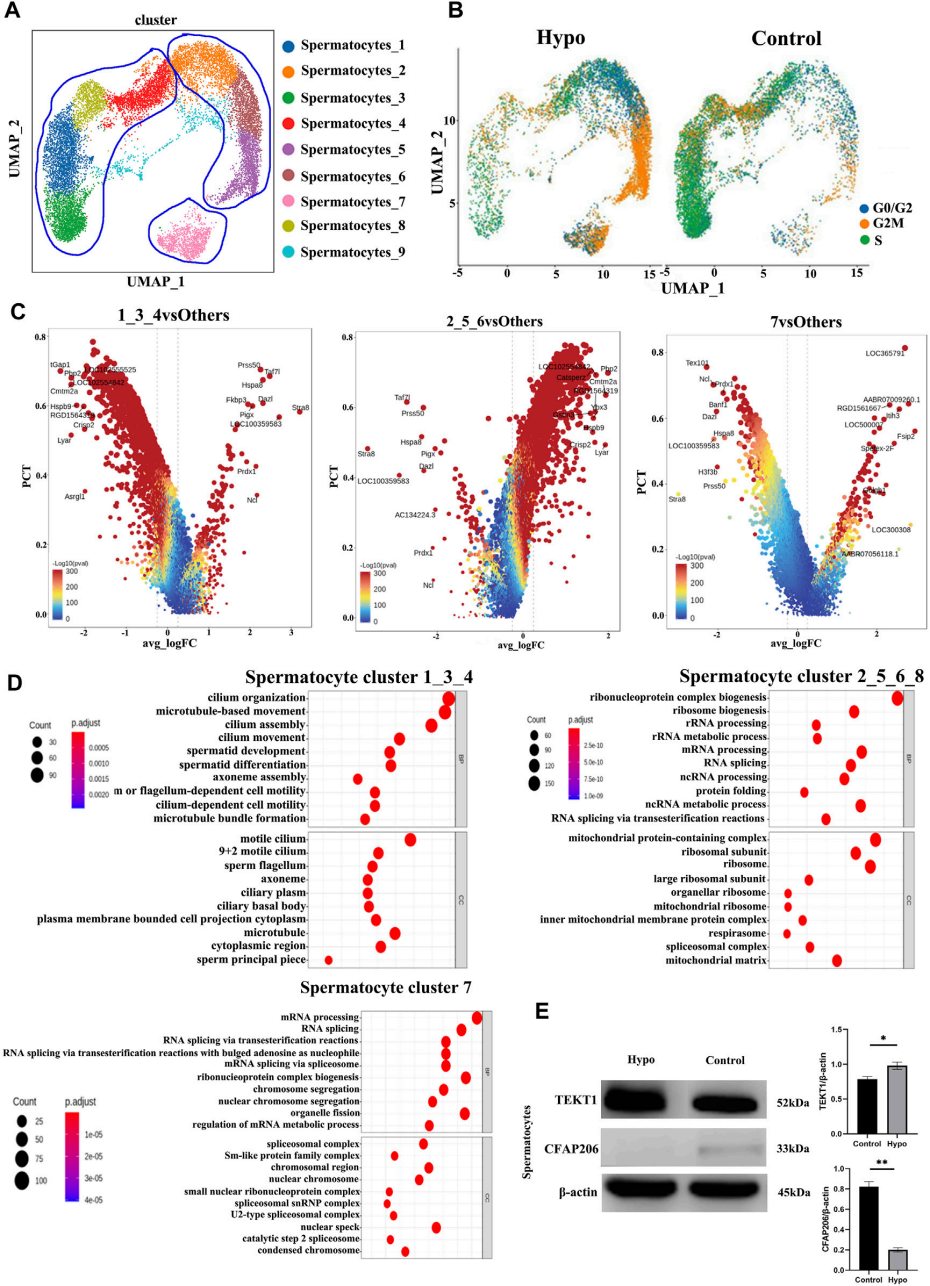
Characteristics of the spermatocyte populations. **(A)** Annotation of spermatocyte subpopulation cell clusters via UMAP visualization. **(B)** UMAP plot of contrast pictures of spermatocyte subpopulation cell cycles. **(C)** Volcano map of spermatocyte subpopulation cell cycles. **(D)** Functional analyses of spermatocyte subpopulation cell cycles. **(E)** Testicular TEKT1 and CFAP206 protein levels were detected by Western blotting.

**FIGURE 5:**
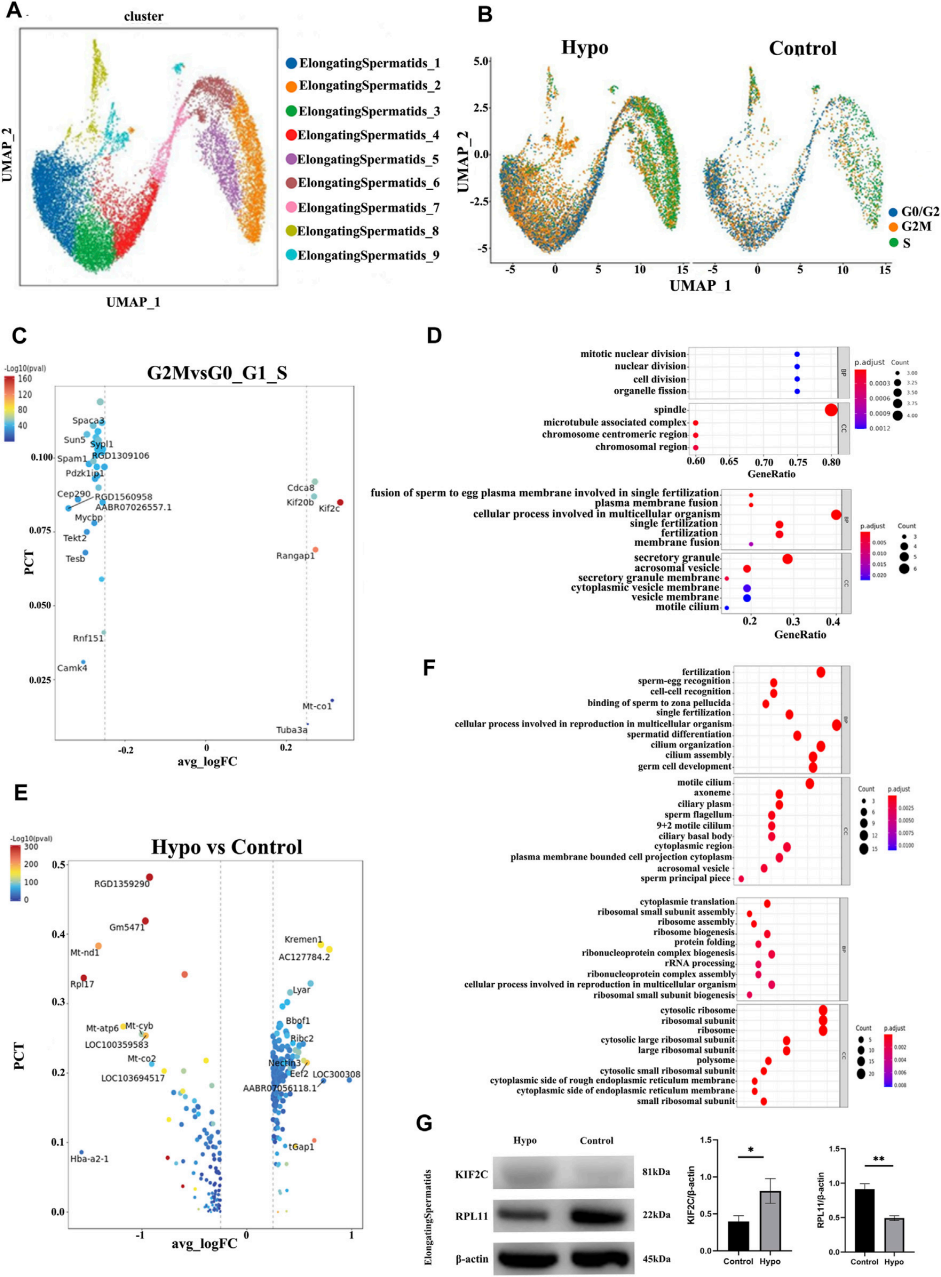
Characteristics of the elongating spermatid populations. **(A)** Annotations of elongating spermatid subpopulation cell clusters via UMAP visualization. **(B)** UMAP plot of contrast pictures of elongating spermatid subpopulation cell cycles. **(C)** Volcano map of elongating spermatid subpopulation cell cycles. **(D)** Functional analyses of elongating spermatid subpopulation cell cycles. **(E)** Volcano map of contrast pictures of elongating spermatids. **(F)** Functional analyses of contrast pictures of elongating spermatids. **(G)** Representative images of Western blotting.

We performed a subgroup analysis to explore the differences in germ cells between the treated and control mouse testes. A UMAP diagram is shown in Figure 4A while an intergroup comparison UMAP diagram is shown in Figure 4B. The subsequent cell cycle analysis demonstrated that most testicular cells from the treated mice were in the G2M phase (Figure 4B). A volcano map divided the spermatocyte subpopulations into clusters 1, 3, 8, and 4; clusters 2, 5, and 6; and cluster 7 (Figure 4C). The GO and KEGG functional pathway enrichment analyses disclosed that clusters 1, 3, 8, and 4 were associated with cilia formation and sperm differentiation, while clusters 2, 5, and 6 were associated with chromosome division and RNA shearing after functional aggregation. Cluster 7 was associated with RNA formation (Figure 4D). Western blotting showed that tektin 1 (TEKT1) was upregulated, while cilia and flagella-associated protein 206 (CFAP206) was downregulated in the spermatocytes of the treated mice. These findings were consistent with those of our analysis. We applied the same technique to analyze the elongating spermatids and discovered that most of the two-cluster population was in the G2 phase (Figures 5A,B). Therefore, elongating spermatids promote maturation toward the G2 phase and increase their functional reserve for sperm–egg binding. A volcano map exhibited both upward and downward genetic adjustments (Figures 5C–E). The GO and KEGG functional pathway enrichment analyses revealed that most of the genes regulating granule secretion and organic matter formation were downregulated, whereas those controlling spindle function were upregulated (Figures 4D–F). Differential functional enrichment between groups showed that the G2 phase of the plateau group induced cilia development, zona pellucida binding, fertilization activity, and other related functions but repressed ribosome-related functions. The WB analysis demonstrated that kinesin family member 2C (KIF2C) was upregulated, while 60S ribosomal protein 11 (RPL11) was downregulated in the testes of the treated mice compared to those of the control (Figure 4G). KIF2C is a biological marker of impaired sperm development, whereas RPL11 is associated with cell cycle arrest. The changes in the expression levels of the preceding genes, following hypobaria hypoxia exposure, may account for the reduced sperm viability observed in response to this treatment.

### 3.4 Pseudotime trajectory analysis determined the state of the germ cells in response to hypobaric and hypoxic exposure during spermatogenesis

**FIGURE 6:**
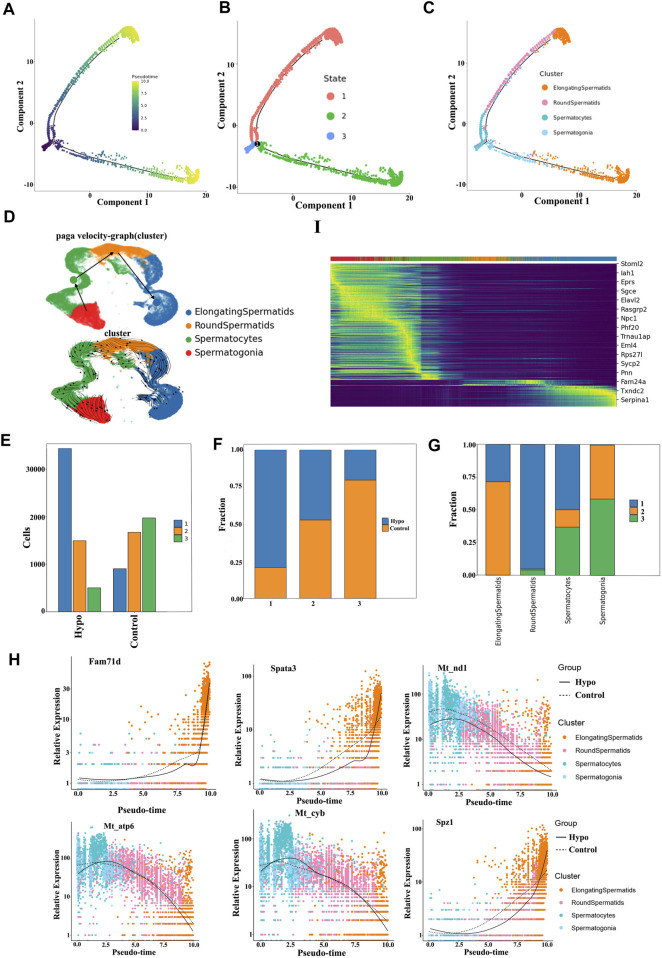
Pseudotime trajectory analysis of testicular germ cell diversity during spermatogenesis. **(A)** Germ cells depicted in a pseudotime trajectory plot. **(B)** Pseudotime trajectory analysis showing germ cells at distinct differentiated states. **(C)** Pseudotime trajectory analysis illustrating germ cell distinction among various cell populations. **(D)** Sperm cell differentiation trajectory. Arrows indicate differentiation of the direction. **(E)** Numbers of testicular cells in each state in the testes of the control and treated mice. **(F)** Proportions of testicular cells in distinct states in the testes of the control and treated mice. **(G)** Cellular components in each state during spermatogenesis. **(H)** Temporal dynamics of key genes along the cell differentiation trajectory. Heatmap of genes presenting significant shear kinetics as differentiation progresses over the latency period. Horizontal coordinates indicate the proposed chronological order, and vertical coordinates represent one gene per row; each column represents the current cell state in average gene expression values, and the colors undergo transition from red to blue.

We conducted a pseudotime trajectory analysis of germ cell maturation during spermatogenesis to investigate the characteristics of the reproductive cells in the testes, following hypobaric hypoxic exposure. We identified three new germ cell states, and these findings aligned with the results of the Monocle 3 analysis (Figures 6A–D). Analysis of the cellular constituents in each state disclosed a parallel relationship between the reproductive cells and their differentiation stages. There were remarkable shifts in the proportion and quantity of germ cells in states 1 and 3 (Figures 6F, G). State 1 comprises spermatogonia, spermatocytes, and cyclic spermatocytes, while states 2 and 3 include elongated and aggregated cyclic spermatocytes. We also analyzed the temporal dynamics of the key genes along the differentiation trajectory. Those associated with specific cellular states changed most significantly along the pseudotime trajectory. Within the germ cell lineage, mitochondrial genes such as *Fam71d*, *Spata3*, and *Mt-nd1* differed in terms of their temporal expression (Figure 6H).

We then plotted a heatmap of the genes presenting significant shear dynamics as differentiation progressed during the latency period. We selected 16 genes that most significantly differed and visualized them in clusters to track the changes in the individual expressed gene modules over the time course. Most of the genes following similar dynamic trends along the trajectory were gradually downregulated. They controlled mitochondrial biogenesis and function, flagellum formation, cell proliferation, and hypoxia-induced apoptosis. Hypoxia alters spermatocyte and long-type sperm cell-cycle arrest. Stomatin-like protein (STOML) controls mitochondrial mediation of cell migration and proliferation, T-cell activation, calcium homeostasis, and cellular stress response. The aforementioned factors may explain why sperm viability decreases after short-term hypobaria hypoxia. Sarcoglycan epsilon (SGCE) was progressively downregulated over time, following hypobaria–hypoxia exposure. Alternative splicing of SGCE exon 8 is regulated in a Sam68-dependent manner, plays a vital role in spermatogenesis and male fertility in mice, and may account for cell cycle arrest in response to hypoxia (Paronetto et al., 2011; Liu et al., 2022).

### 3.5 Interactions among cells in the testis

**FIGURE 7:**
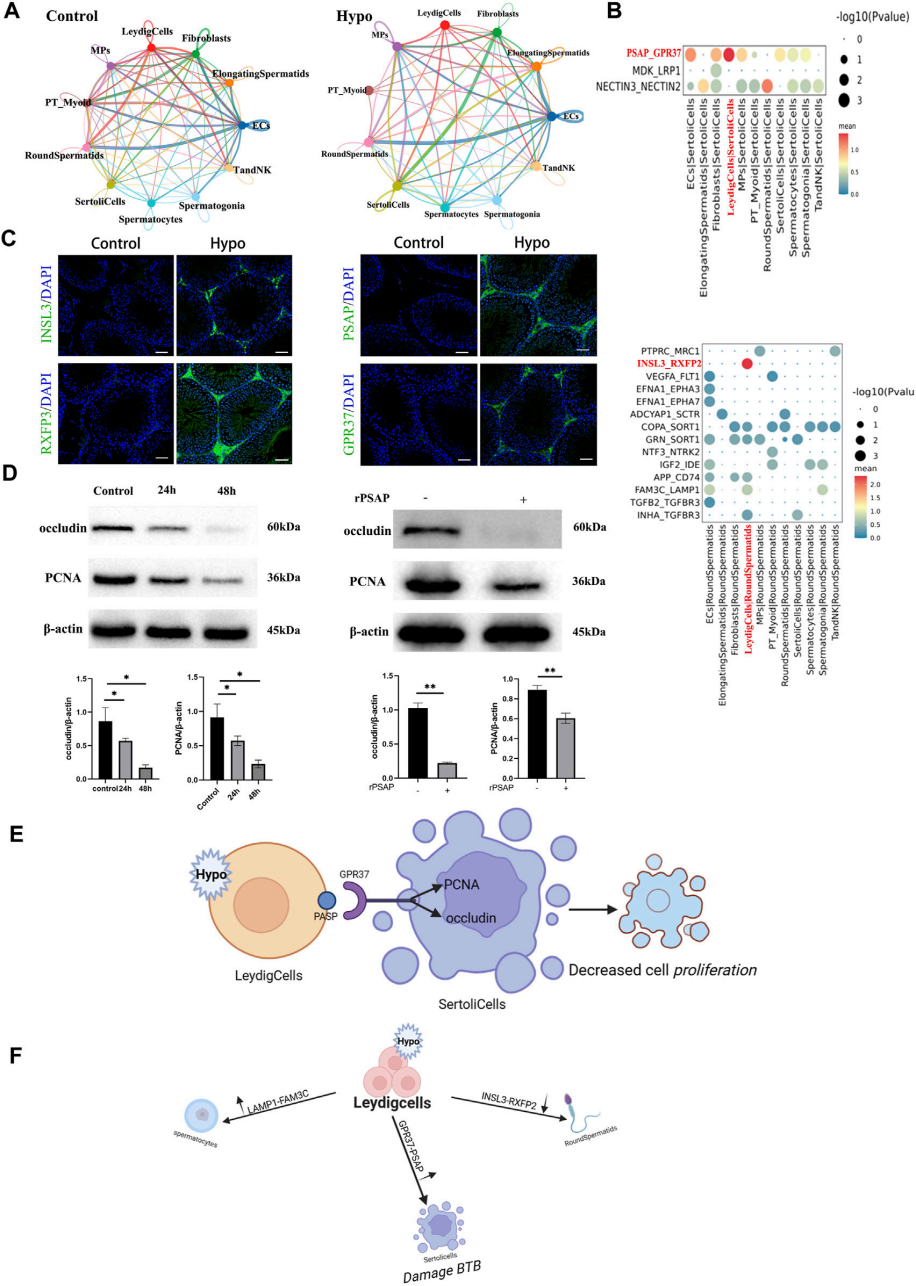
Communications between sperm and other cells. **(A)** Network diagram of ligand–receptor interactions. **(B)** Bubble plots of ligand–receptor interactions showing expression intensity and specificity in each cell type. **(C)** Immunofluorescence staining of frozen testis sections. Scale bar = 100 μm. **(D)** Sertoli cells were cultured under 1% (v/v) O_2_ for 24 h and 48 h, respectively, and the expression levels of the relevant proteins were measured. Sertoli cells were incubated with 500 ng/mL rPSAP for 30 min, and their occludin and PCNA expression levels were examined by Western blot. β-actin was the loading control, *p < 0.05, **p < 0.01. **(E)** Schematic diagram showing the potential mechanism inducing PSAP-GPR36 interactions in Sertoli cells. **(F)** Summary of hypoxia-induced communication in Sertoli cells.

Ligand–receptor interactions stabilize cell–cell communication. The most critical component of germ cell development is somatic cell-mediated regulation of the testicular microenvironment. CellPhoneDB inferred cell–cell communication within each subtype based on receptor–ligand interactions. It also identified ligand–receptor pairs and molecular interactions for each major cell type. Overall, we systematically analyzed potential cell–cell interactions based on the co-expression of known ligand–receptor pairs within any pairwise combinations of Sertoli cells, Leydig cells, endothelial cells, and macrophages. We compared interactions between principal and non-principal cells. For the control, there were strong interactions between Leydig cells and round spermatids. For the hypobaria hypoxia treatment group, there were strong interactions between Sertoli cells and Leydig cells. However, the interactions among fibroblasts, elongated spermatocytes, and spermatogonia significantly differed. A heatmap of the subgroup analysis revealed that cell communication was significantly enhanced between round spermatids 5 and elongated spermatocytes 2, between round spermatids 5 and round spermatids 4, and between round spermatids 5 and spermatocytes 5 in response to hypobaria hypoxia exposure. Bubble plots of the ligand–receptor interactions disclosed significant changes in testicular cell communication for the mice exposed to hypobaria hypoxia compared to that of the control mice (Supplementary Figures S1A–S2D). We then compiled datasets for stress- and hypoxia-related genetic interactions. Figure 7C shows that communication routes significantly differed between the Leydig cells and the round spermatids because of the relative differences in the insulin-like peptide 3 (INSL3)–relaxin family peptide receptor 2 (RXFP2) interactions within them. The aforementioned reciprocal pair may co-regulate urogenital tract development and female fertility. Mutations may result in cryptorchidism (failure of the testes to descend from the abdomen into the scrotal sac) (Andretta et al., 2023). Prosaposin (PSAP)-G protein-coupled receptor 37 (GPR37) interactions enhance Sertoli–Leydig cell communication. PSAP may mediate sperm–egg interactions, while Sertoli cells postnatally express both GPR37 and its ligand PSAP. Sertoli cell proliferation and maturation were suppressed in Gpr37-null mutant mice undergoing postnatal testicular development (Magargee et al., 2000; La Sala et al., 2015). The family with sequence similarity 3 metabolism-regulating signaling molecule C (FAM3C)–lysosomal-associated membrane protein 1 (LAMP1) interaction regulates spermatocyte, endothelial cell, Leydig cell, and spermatogonia functions. LAMP1 may be associated with the cytoplasmic vesicles rather than the developing acrosome (Moreno, 2003). Mesenchymal and Sertoli cells communicate with GPR37 via its ligand PSAP. Here, we confirmed the participation of PSAP-GPR37 in hypoxia-induced cell proliferation and demonstrated that the protein levels of proliferating cell nuclear antigen (PCNA) and occludin were lower in hypoxia-exposed cells than those in untreated testicular cells. Moreover, when the latter was incubated with recombinant prosaposin (rPSAP), they presented occludin and PCNA downregulation.

## 4 Discussion

Environmental perturbations may induce epigenetic modifications in the germline, thereby disrupting spermatogenesis and causing infertility. Hypoxia alters the expression of testicular genes and, therefore, interferes with spermatogenesis (Li and Yang, 2022). Here, single-cell sequencing identified a cluster of hypobaric hypoxia-specific genes. This discovery clarified the distribution of these genes within spermatogenesis and improved our understanding of the mechanisms by which testicular function is maintained. We also examined the RNA modification levels in the testes of mice exposed to hypoxia. The findings made herein could provide insights into the possible impact of hypobaria hypoxia on human testes and showed that hypoxia impairs spermatogenesis in individuals exposed to it (Bai et al., 2018). Hypoxia damages PT myoids, arrests spermatocytes in the S phase, and causes elongating spermatids to shift toward the G2 phase as they mature. This shift augments their functional reserve for sperm–egg binding. The preceding observations indicate that activation of the TNF-α/TNFR I-mediated extrinsic apoptosis pathway may play a pivotal role in hypoxia-induced spermatogenesis impairment. However, the extent of communication among different types of testicular cells has not been thoroughly investigated. Our research revealed that hypobaric hypoxia upregulates TEKT1 and KIF2C, and downregulates CFAP206 and RPL11. KIF2C is a mitotic-centromere-associated kinesin. This microtubule-dependent molecular motor protein promotes mitotic segregation (Ritter et al., 2015). Hence, KIF2C expression is a potential sperm damage biomarker. The quantity of KIF2C within the sperm is closely associated with fertilization and the early stages of embryogenesis. Animal experiments demonstrated the importance of KIF2C in spermatic mitosis and meiosis (He T. et al., 2022). We found that environmental stimuli upregulate KIF2C in elongated spermatids, thereby influencing spermatic mitosis and normal sperm cell development. The conserved ribosomal protein complex consisting of 5S rRNA, RPL5, and RPL11 might play a vital role in the nucleolar stress response of mammalian cells (Donati et al., 2013). Ribosomal stress causes the complex to dissociate, strengthens the interaction between the RPL5/RPL11 complex and mouse double minute 2 homolog (MDM2), inhibits MDM2 E3 ligase, promotes p53 transcription, and arrests the cell cycle in the G1/S and G2/M phases. Ionizing radiation (IR)-induced apoptosis in spermatogonia may be linked to RPL23a downregulation (Li et al., 2020; He Y. X. et al., 2021). In turn, the latter attenuates binding between RPL23a and RPL11, and indirectly activates the RPL11-MDM2-p53 pathway, which triggers p53 and, by extension, apoptosis. In this study, RPL11 downregulation in the hypobaria hypoxia-exposed group may have weakened the binding between RPL23a and RPL11, indirectly activated p53, suspended the cells in the G2 phase, and caused them to undergo apoptosis. CFAP206 participates in the axonemal radial spoke assembly and may be implicated in cilium motility (Paronetto et al., 2011). CFAP206 is essential for the sperm flagellar axoneme assembly and stabilization. Dual allelic mutations may result in flagellar malformations by causing morphological and functional defects of the sperm flagellum and, therefore, early embryonic dysplasia and male mouse sterility (Wang et al., 2022). TEKT1 is involved in the nucleation of mature sperm flagellar axonemes and may be crucial in the substrate assembly (Larsson et al., 2000). Alterations of the genes linked to sperm flagella formation may result in aberrant sperm flagellum morphology and, therefore, reduced sperm motility (Shen et al., 2019). The results of the present work suggest that hypoxia exposure can lead to abnormal sperm flagellum morphology and function. The interaction between CFAP206 and CSC-RS plays a critical role in the formation of complexes with CFAP251/WDR66 or CFAP91, which are required for the sperm flagellar axoneme assembly and stabilization. Dual allele mutations in CFAP206 may create morphological and functional defects in sperm flagella, lead to early embryonic dysplasia, and contribute to male mouse infertility (López-Jiménez et al., 2022). Thus, CFAP206 is vital for the maintenance of the sperm flagellum structure and function. In contrast, CFAP206 promotes elongated spermatocytes to the G2 phase and activates spindle function. Wdr62-deficient spermatocytes present intermediate phase I arrest and undergo the abnormal spindle assembly. Wdr62 deficiency leads to centrosomal protein 170 (CEP170) downregulation, abnormal spindle assembly, and developmental arrest (Qin et al., 2019). Based on the preceding findings, we hypothesize that variations in the foregoing genes may partially explain aberrant sperm development.

The testis is a highly complex gland comprising Leydig, Sertoli, germ, and peritubular myoid cells, as well as extracellular matrix (ECM) material (Li et al., 2023). Interactions among these cells are crucial for correct ECM remodeling, as well as several developmental and morphogenetic processes (Piprek et al., 2019). Exploration of these intercellular communication networks may help clarify the effects of hypoxia and stress on various tissues and organs. The results of this work indicated that hypoxia and reciprocal stress-related gene pairs can mediate intercellular communication in the testis. Single-cell RNA sequencing of gonadal tissue enables us to perform pseudotime trajectory analyses and identify novel cellular differentiation states. Here, we analyzed the germ cells implicated in spermatogenesis and discovered three new cellular states. Hypoxia exposure significantly altered the proportions and quantities of germ cells in states 1 and 3. It also significantly altered certain new signals. The mitochondrial genes exhibited distinct temporal expression patterns. The dynamic trends of DEGs showed that most of them were gradually downregulated, and they controlled mitochondrial biogenesis and function, cilia formation, cell proliferation, apoptosis, and hypoxia regulation. Hypoxia exposure may alter spermatogonia and/or arrest the cell cycle in elongated spermatids. Differential dynamic expression of lineage-specific genes might help explain the observed genetic heterogeneity in the testes, following hypoxia exposure. However, our current understanding of the inhibition of male reproduction by hypoxia remains incomplete. Future investigations should aim to determine the extent to which these putative mechanisms are invoked to enable cellular interactions. Subsequent studies should also endeavor to establish why hypoxia reduces the relative number of endothelial cells (Figure 2C) and whether this phenomenon affects sperm vitality.

## 5 Conclusion

Overall, the present study provided empirical evidence that hypoxia exposure damages PT myoids and arrests spermatocytes in the S phase. It also showed that it causes elongating spermatids to advance toward the G2 phase during maturation. To the best of our knowledge, the present study is one of the first to elucidate, at single-cell resolution, the molecular mechanism and regulatory signaling associated with the testicular toxicity of hypobaric hypoxia exposure. We believe that it offers a novel perspective of the regulatory pathways involved in reproductive toxicity induced by exposure to physical hazards in the ambient environment.

## Data Availability

The datasets presented in this study can be found in online repositories. The names of the repository/repositories and accession number(s) can be found below: https://ngdc.cncb.ac.cn/, PRJCA020831.
