# Supplementary figures and images for: Cascading effects of hypobaric hypoxia on the testis: insights from a single-cell RNA sequencing analysis
Source: Front Cell Dev Biol. 2023 Nov 15;11:1282119. doi: 10.3389/fcell.2023.1282119 (PMC10684926; doi:10.3389/fcell.2023.1282119)

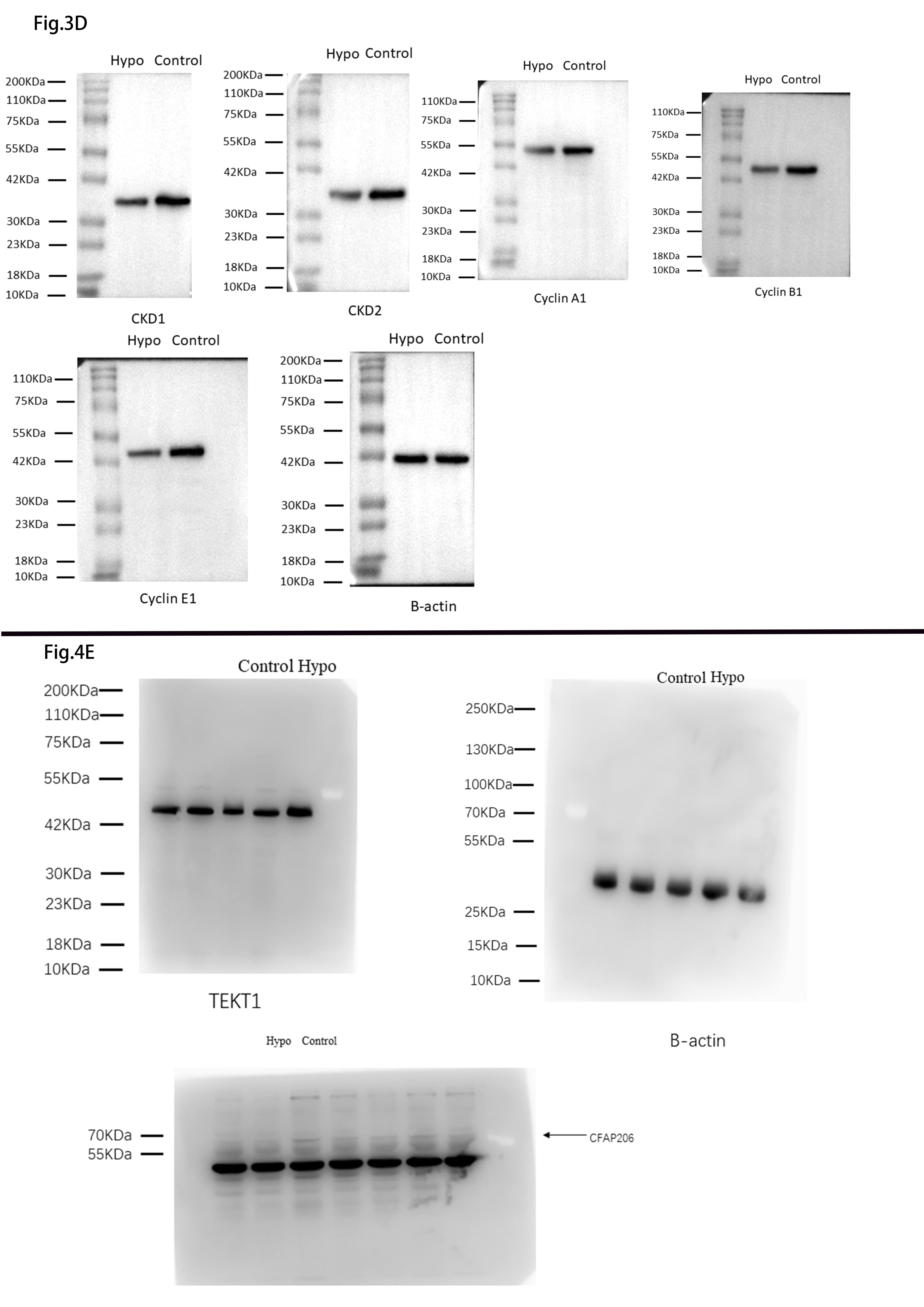

Supplement: Supplementary file 1 [file Image1.TIF]

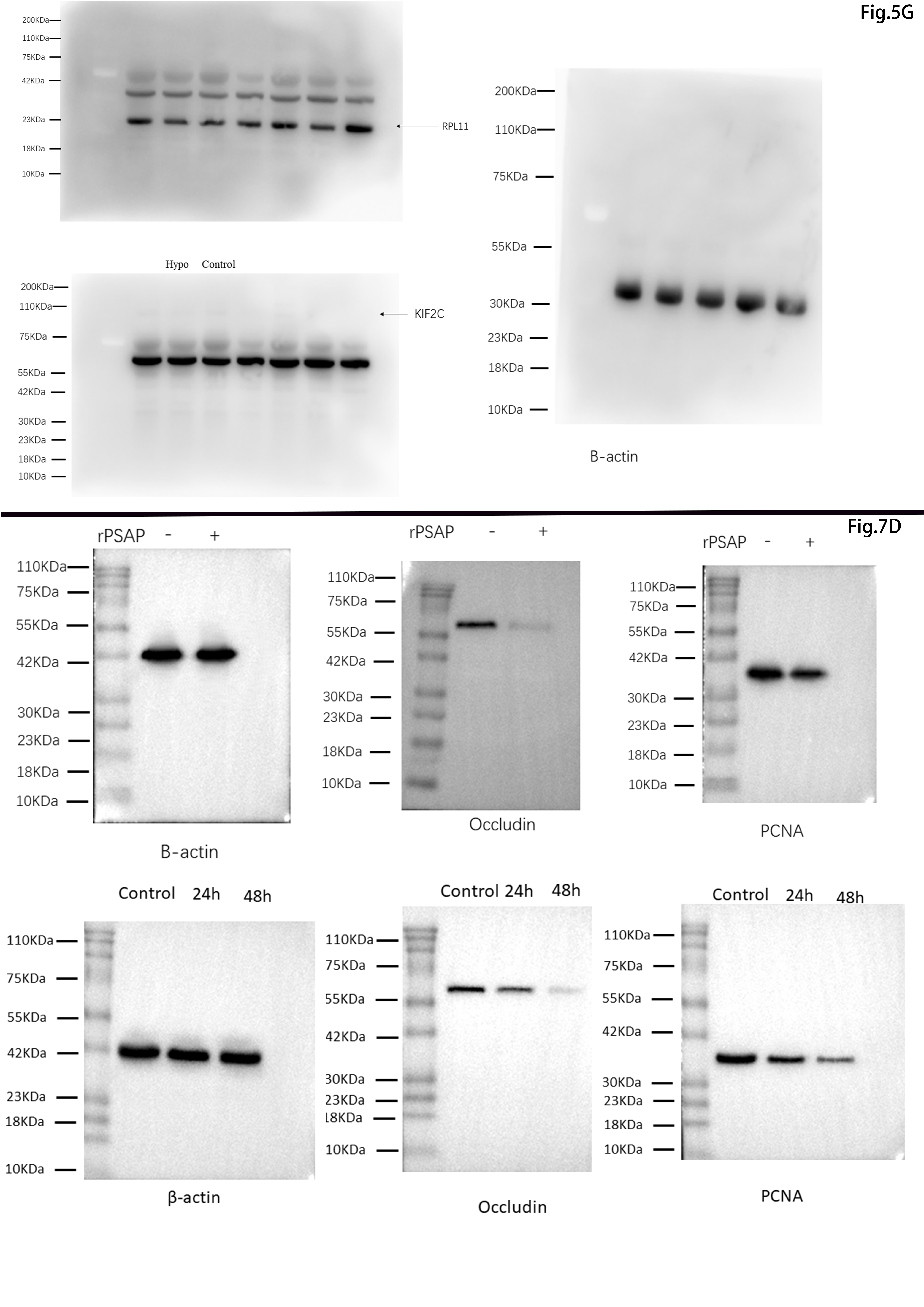

Supplement: Supplementary file 2 [file Image2.PNG]
