# Supplementary material for: Cascading effects of hypobaric hypoxia on the testis: insights from a single-cell RNA sequencing analysis
Source: Front Cell Dev Biol. 2023 Nov 15;11:1282119. doi: 10.3389/fcell.2023.1282119 (PMC10684926; doi:10.3389/fcell.2023.1282119)
Supplement: Supplementary file 3 [file DataSheet1.docx]

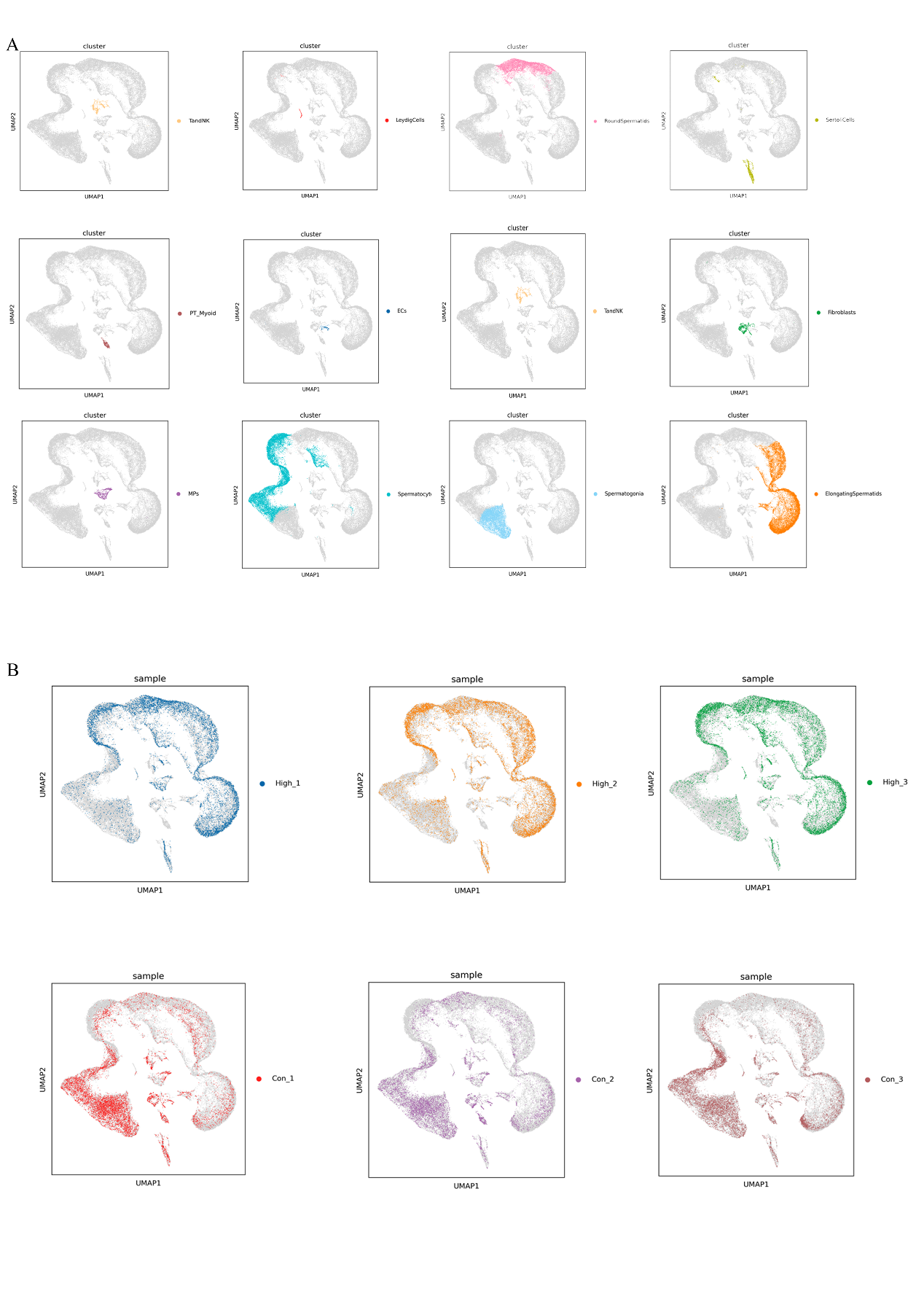


**Fig. S1**. Identification of cell types about testis. **A** Cell type identification about hypobaric hypoxic exposure in testis. **B** Revealed excellent repeatability


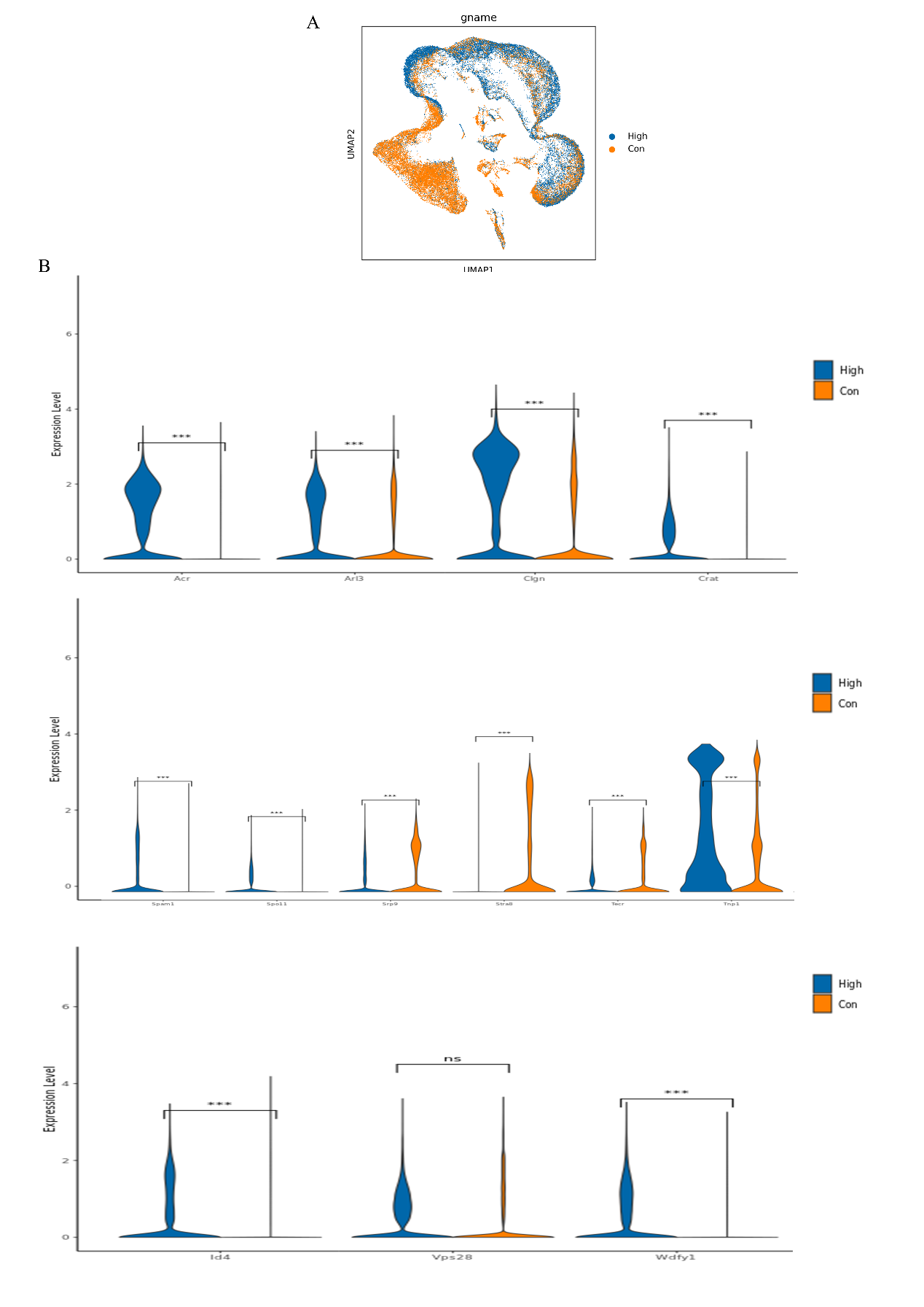


**Fig. S2.** UMAP and differential gene expression plot **A**. The UMAP graph colored by group. **B**. Distribution of low-pressure hypoxia differential genes reported in the previous literature in single-cell data, with differences in expression between groups.

**
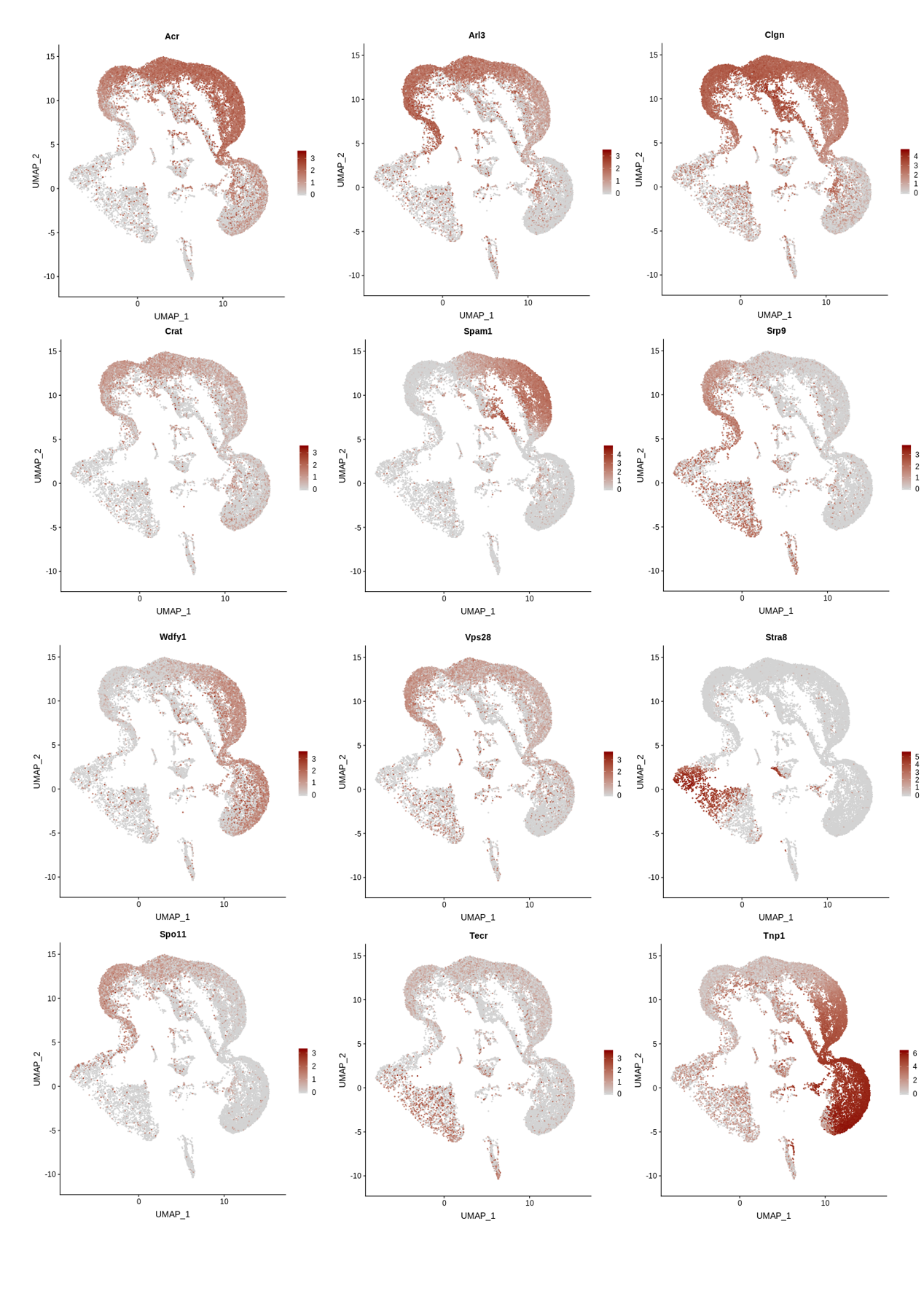
**

**Fig. S3.** UMAP diagram of low-pressure hypoxia genes. The differential genes were mainly derived from different sperm cells.


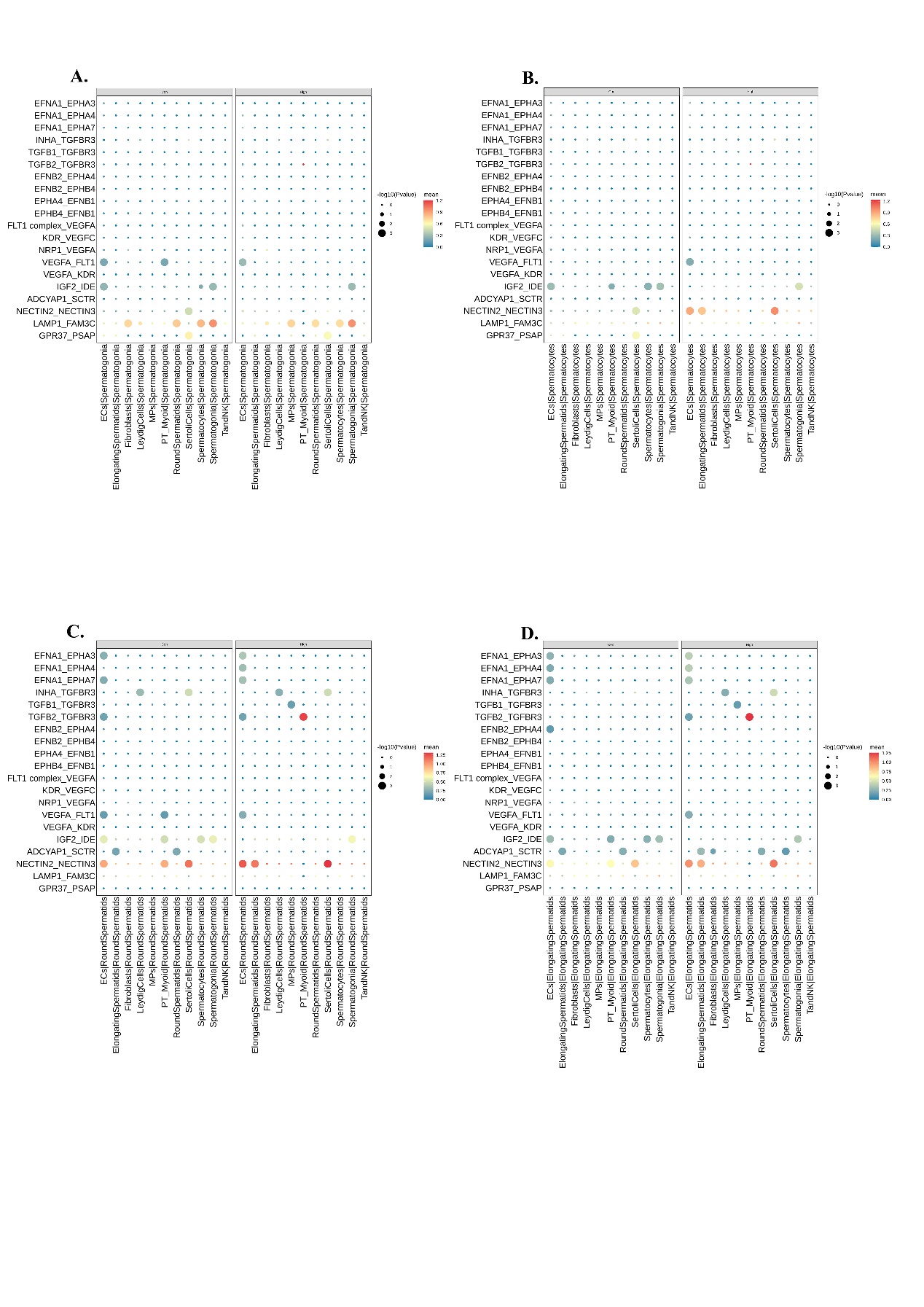


**Fig. S4.** Cell–Cell communication about sperm cells during hypobaric hypoxic exposure. **A**. Communication of spermatogonia with other testicular cells. **B**. Communication of Spermatocytes with other testicular cells. **C**. Communication of round spermatids with other testicular cells. **D**. Communication of elongating spermatids with other testicular cells.


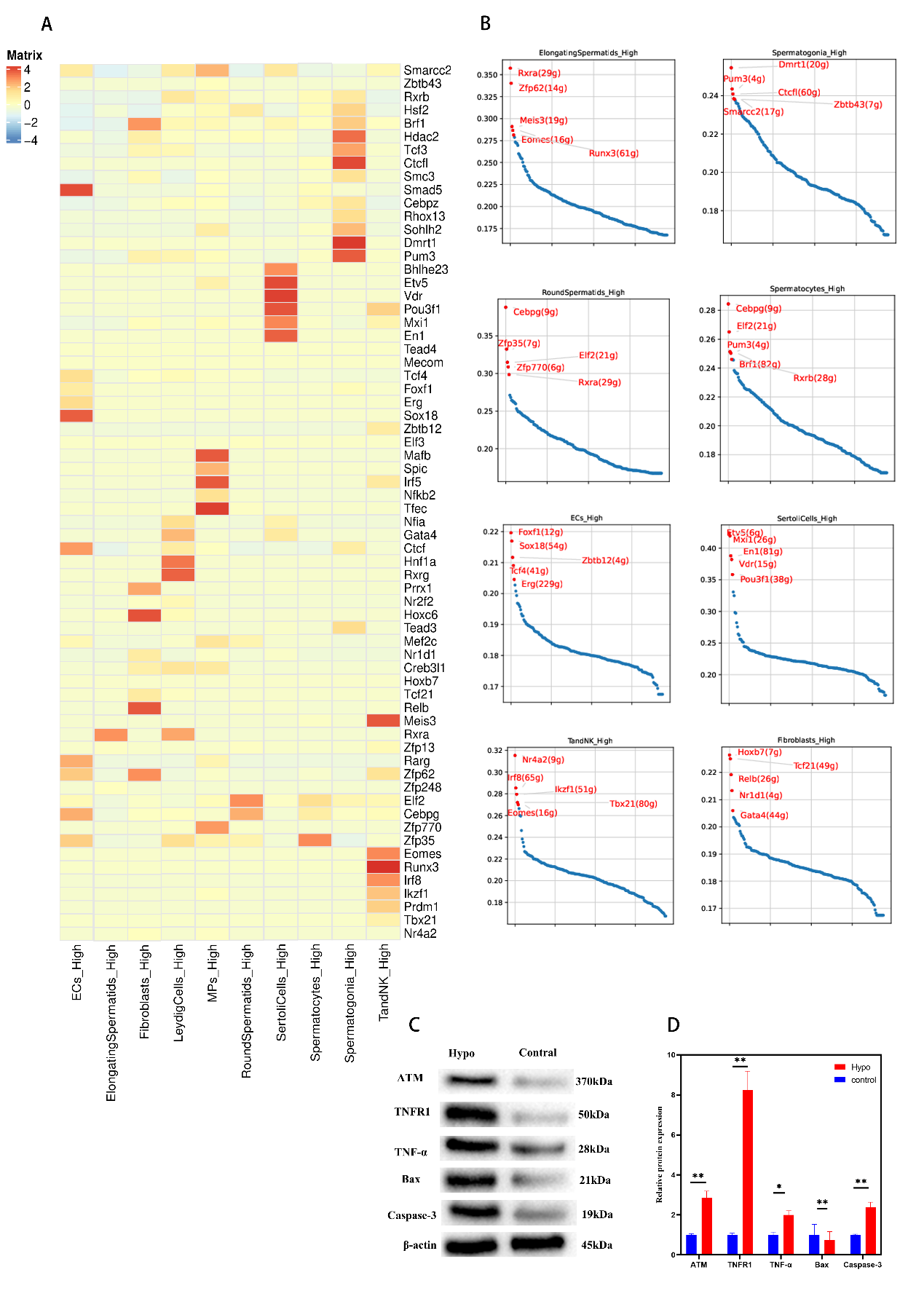


**Fig. S5.** Heatmap of transcription factor (TF) activities in each cell type and the protein expressions of ATM/p53 and TNF-α/TNFR Ⅰ-mediated signaling pathways. A. UMAP Uniform Manifold Approximation and Projection for each cell type. B. Scatter plots of regulon specificity for each cell type, highlighting the most high top regulon. C. The protein expressions of ATM, Bax, TNF-α, TNFR Ⅰ, and Caspase-3 were detected in the testis of control group and treated group D. Densitometric analysis was performed on these protein bands. Data were homogenized. n = 3, *P< 0.05, **P< 0.01compared to control.
